# Child welfare services and social media – childhood, being and becoming in a digital society

**DOI:** 10.3325/cmj.2018.59.90

**Published:** 2018-04

**Authors:** Siv-Britt Björktomta, Heidi Aarum Hansen

**Affiliations:** 1 Centre for Social Work (CESAR), Department of Sociology, Uppsala University, Uppsala, Sweden *siv-britt.bjorktomta@soc.uu.se*; 2 Department of Health and Welfare Studies, Østfold University College, Østfold, Norway

Different arenas in which children reside provide specific conditions for children’s development. Behavior in the home and school arenas is governed by adults’ norms, while on the internet children have more space to design norms of their own. The internet use continues to increase and the young generation uses social networks on a daily basis. Research shows that children develop internet skills long before their ninth birthday (1,2). Moreover, they are often more familiar with social media than adults around them. These changes impose new requirements for child welfare professionals and create the need for new knowledge in the field of social work. At the same time, children´s involvement in the digital arena challenges the discourse on service provision in the public sector.

## Social media – pleasures and worries

Smartphones, broadband, and visual applications enable children and young people to obtain and share information in new ways, which is why these modalities should be included in the assessments of children’s psychosocial health and environment (3). Swedish surveys show that 90%-95% of children aged between 12 and 18 use different types of social media, such as chat, text messages, or email (2,4). Social media facilitate communication and promote sharing experiences and feelings through words and pictures. The young generation’s daily life on the internet is mainly ordinary and undramatic; they use the internet and mobile phones to socialize with friends but also to manage intimate relationships. However, children are a particularly vulnerable group when it comes to violence, both on the internet and elsewhere. The Swedish child rights organization called Bris (its full name translated into English means “Children’s Rights in the Society”) organizes channels where children can seek support, free of charge and anonymously via email and chat (5). Bris publishes children and young people´s everyday-life stories, mainly focusing on threats and abuse online, conflicts in the family, anxiety, mental illness, bulling, and sexual violence (5). Young people also often pose questions about personal integrity and handling security and online protection.

The digital context requires social workers to possess types of knowledge different from those needed in face-to-face communication. For example, professionals need to be familiar with the jargon, language and communication patterns used by children (6). It has been shown that young people believe that they can get help more easily if social workers use text messages and Facebook (6). Moreover, for them this is an easier way to establish the first contact and to build trust in the real life. Consequently, we believe that social workers need to venture into the new internet territory to a greater extent than before.

## Children as agents

A useful theoretical paradigm that can help understand children’s social media-related behavior is sociology of childhood. It criticizes the developmental psychology perspectives that prevailed in the past. Psychology perspectives view the child as an innocent object under (natural) development (7,8), claiming that all children develop similarly at the same age, regardless of the conditions they grow up in (8,9). In contrast, sociology of childhood perceives children as *agents*, with their own interests, independent of those of adults (10-12).

The key concepts of sociology of childhood are being and becoming. While the “being child” is seen as a social actor actively constructing “childhood,” the “becoming” child is seen as an “adult in the making,” lacking competencies of the adult that he or she will “become” (13). In other words, the internet can be perceived as another arena in which the child as a competent agent gains a specific meaning. Through real time communication in social media, eg, instant messaging, the child is literally “being.” Understanding the child as “being” also corresponds with social workers’ view of children. Social workers prefer to involve the child in assessment work and determining measures, as opposed to the deeply rooted view of children as being unable to make decisions and in need of protection. As a consequence, professionals struggle when it comes to children´s participation and are often unable to involve children (14,15), be it because of organization’s framework conditions or professionals’ inability to talk with children. This makes the sociology of childhood’s idea to comprehend the child as a subject problematic in practical work. Taking children seriously requires discussing the child’s best interests based on the child’s *own* story.

## Protecting children

The child welfare services are designed based on children’s needs and the idea of what children are. But this is not a fixed concept. Actually, the cultural understanding of childhood is multifaceted and relative. James and James (11) use the concept *cultural politics of childhood*, meaning that childhood is constructed by interaction of cultural conditions, political mechanisms, and the discourses they create, functioning in ways that both widen and limit children's spaces of actions.

In the Nordic countries, the social services have a unique position with regard to family politics and are given by the legislation a sweeping authority to intervene in cases of children’s exposure to neglect (15). The United States of America and Northern European countries adopted a similar approach and have recently introduced reforms focused on the protection of children. Although it is difficult to compare different contexts, historical backgrounds, and social welfare systems, we can see that this approach becomes an international trend (16).

Protecting children involves professionals’ understanding of new communication patterns. Although social media enable children to share experiences and feelings, the child’s need for care and protection emerges as an important factor in the development of a good psychosocial environment and health. However, there is also the risk of overestimating the child’s competence. Comprehending the child as an active subject does not exclude his or her need for protection and support in a difficult life situation (17).

## Children´s best interest – even on the internet

In conclusion, the child´s best interests and needs change over time and contexts, and cannot be defined once and for all. The society´s idea of the child and adults’ behavior toward children influence how children see themselves and how they interact with others. Although the welfare state of today promotes children’s participation, children’s impact is limited to formal and bureaucratic procedures and language (17,18).

In the internet arena, children have more space for action. Accordingly, their use of social media challenges the public services. Children´s navigating social media landscapes is an example of a change that calls for new research into the following questions: What do social workers think about contacting children via social media? Can social media be used as a tool in case assessment? And can the internet enable social workers to reach children in difficult life situations?
